# Effects of psilocybin on personality, psychiatric symptoms, and values: Exploring mediating effects of the acute psychedelic experience

**DOI:** 10.1177/02698811251408769

**Published:** 2026-01-26

**Authors:** Jess Kerr-Gaffney, Samuel Myrtle, Famia Askari, Catherine Bird, Nadav Liam Modlin, Allan H. Young, James Rucker

**Affiliations:** 1 Department of Psychological Medicine, Institute of Psychiatry, Psychology & Neuroscience, King’s College London, UK; 2 School of Clinical Medicine, University of Cambridge, UK; 3 Division of Psychiatry, Imperial College London, UK; 4 South London and Maudsley NHS Foundation Trust, UK

**Keywords:** psychedelic, psilocybin, personality, values, cognitive flexibility

## Abstract

**Background:**

Changes in well-being, personality, and personal values have been documented post-psilocybin; however, evidence from placebo-controlled trials is limited.

**Aims:**

To examine the effects of psilocybin versus placebo on psychiatric symptoms, personality, and personal values in healthy participants. Potential mediators were also explored.

**Methods:**

This secondary analysis used data from a phase I, double-blind, randomised, placebo-controlled trial testing a single dose of 10 mg (*n* = 30) or 25 mg psilocybin (*n* = 30) versus an inert placebo (*n* = 29) in 89 healthy participants. Effects of psilocybin on personality (Neo Five-Factor Inventory; NEO-FFI), psychiatric symptoms (Symptom Checklist-90; SCL-90), and values (Life Changes Inventory; LCI) at short- (day 8) and long-term follow-up (day 85) were analysed using mixed-effects models. Group differences in cognitive flexibility (Intra-Extra Dimensional Set Shift task; IED) at day 8 were analysed using a Kruskal–Wallis test. Potential mediating effects of the acute psychedelic experience (Five-Dimensional Altered States of Consciousness Questionnaire; 5D-ASC) were explored.

**Results:**

No between-group differences were found on the NEO-FFI, SCL-90, or IED. Both psilocybin groups showed greater LCI absolute change scores at both follow-up points compared to placebo. The 5D-ASC oceanic boundlessness subscale partially mediated these changes. Oceanic boundlessness also fully or partially mediated differences across several LCI subscales, and auditory alterations mediated differences on one subscale.

**Conclusions:**

The acute psychedelic experience, namely oceanic boundlessness and, to a lesser extent, auditory alterations, mediates self-reported changes in values in healthy volunteers. Findings from this exploratory study are tentative and should be replicated in larger samples.

## Introduction

Psilocybin (4-phosphoryloxy-N,N-dimethyltryptamine) is a naturally occurring tryptamine and classic psychedelic, producing acute perceptual and mood alterations in humans, in part through partial agonism of 5HT_2A_ receptors (Madsen et al., 2020). Psilocybin and other classic psychedelics can facilitate changes in beliefs, values, and subjective well-being, alongside mystical and spiritual experiences (Forstmann et al., 2020; Griffiths et al., 2006, 2018; Nayak et al., 2023; Timmermann et al., 2021). In the past decade, there has been a resurgence of early-stage trials testing safety and efficacy of psilocybin to treat a range of mental health conditions, including depression and anxiety in advanced-stage cancer (Griffiths et al., 2016; Grob et al., 2011; Ross et al., 2016), major depressive disorder (MDD; Carhart-Harris et al., 2021; Goodwin et al., 2022), alcohol use disorder (Bogenschutz et al., 2022), obsessive compulsive disorder (Moreno et al., 2006), and anorexia nervosa (Peck et al., 2023).

The subjective effects of psilocybin are thought to primarily involve agonism of the 5-HT_2A_ receptors, with downstream changes in gene expression (López-Giménez and González-Maeso, 2017). Modulation of other 5-HT receptors, as well as indirect effects on glutamatergic systems, is also contributory. Psilocybin alters neural circuitry, promoting neural ‘plasticity’ in animal models (Calder and Hasler, 2023). However, the mechanisms via which these neurochemical and structural changes might facilitate improvements in psychiatric symptoms, as well as personally transformative experiences, are not currently well characterised.

Perhaps the most mentioned mediator of psychedelic response is the quality of the acute subjective experience, including ‘ego-dissolution’, mystical-type experiences, and ‘unitive’ states. For example, mystical-type experiences during psilocybin administration have been shown to facilitate long-term changes in trait openness in healthy volunteers (MacLean et al., 2011). Similarly, mystical experiences and intensity of the acute experience during administration of lysergic acid diethylamide (LSD) are associated with long-term changes in well-being and life satisfaction in healthy volunteers (Schmid and Liechti, 2018). In a small sample of patients with treatment-resistant depression (TRD), insightfulness and spiritual experience have been shown to correlate with changes in trait neuroticism and extroversion (Erritzoe et al., 2018), whilst high oceanic boundlessness and low anxious ego-dissolution have been associated with changes in symptoms of depression (Roseman et al., 2018). In the largest randomised controlled trial (RCT) to date using psilocybin for TRD, oceanic boundlessness, visual restructuralisation, and emotional breakthrough during treatment were found to be most strongly related to reductions in depression scores 3 weeks post-treatment (Goodwin et al., 2025). Similar findings have been replicated outside of clinical trials, with psychedelic users reporting stronger mystical-type experiences during administration, also reporting more positive changes in well-being (Haijen et al., 2018).

Other factors that have been suggested to mediate outcomes include mindfulness, insight, and cognitive flexibility (Kočárová et al., 2021). Cognitive flexibility refers to the ability to shift perspective or behaviour in response to changes in the environment. It is hypothesised that psilocybin and other classic psychedelics create a cognitive state that may improve therapeutic intervention by relaxing prior beliefs and enhancing capacity for change and flexible thinking (Torrado Pacheco et al., 2023). Indeed, poor cognitive flexibility is a prominent feature of a range of psychiatric disorders that have shown a positive response to psychedelics, including depression, anorexia nervosa, and substance use disorder (Kanen et al., 2019; Murphy et al., 2012; Tchanturia et al., 2012). Psilocybin improves cognitive flexibility in animal models (Conn et al., 2024; Torrado Pacheco et al., 2023) and in patients with depression (Doss et al., 2021), although changes in the latter group were not related to improvements in depression. Similar findings have been reported after ayahuasca use (Murphy-Beiner and Soar, 2020).

Evidence for mechanisms of response to psychedelics discussed thus far mostly derives from open-label, uncontrolled studies. This secondary data analysis aimed to examine short- and long-term effects of 10 or 25 mg of psilocybin compared to placebo on psychiatric symptoms, personality, and personal values in healthy volunteers, and explore the potentially mediating roles of the acute psychedelic experience and cognitive flexibility.

## Methods

### Design

This study represents secondary data analysis of a phase I, double-blind, randomised, placebo-controlled trial investigating the safety of a single dose of 10 or 25 mg psilocybin compared to placebo in 89 healthy participants, conducted at King’s College London (EudraCT number: 2018-000978-30). Participants were followed up over 12 weeks. The trial was funded and sponsored by COMPASS Pathways. Further details of the trial and the primary analyses are presented in Rucker et al. (2022).

### Participants

Inclusion criteria were adults aged 18–65 years old, proficient in English, and able to complete assessments without assistance. Exclusion criteria included no current or history of a psychiatric disorder, first-degree relative with a history of a psychiatric disorder, psilocybin use within 1 year prior to enrolment, psychiatric medication in the past year, pregnancy, nursing, or planned pregnancy in female participants, cardiovascular conditions, uncontrolled or insulin-resistant diabetes, seizure disorder, positive urine drug screen for illicit drugs, participation in another investigational drug or device trial, abnormal or clinically relevant results on physical examination, bloods, or ECG at screening or any other clinically relevant illnesses deemed by the investigator to have potential to interfere with interpretation of study results or put the participant at risk.

The trial was conducted in accordance with the Declaration of Helsinki and International Conference on Harmonisation guidelines for Good Clinical Practice (ICH-GCP). The study received a favourable opinion from the London-Brent Research Ethics Committee (18/LO/0731). All participants gave written informed consent prior to eligibility screening.

### Procedure

Potential participants attended a screening visit at which eligibility was determined, and consent was taken. Eligible participants then attended a baseline session (day 0), 1 day before the anticipated drug administration visit. Baseline variables were assessed, eligibility re-checked, and participants took part in a group preparatory session.

During the study drug administration visit (day 1), participants were randomised 1:1:1 using blocked randomisation to receive 10 mg psilocybin, 25 mg psilocybin, or a placebo. The study drug was COMP360, COMPASS Pathways’ proprietary pharmaceutical-grade synthetic psilocybin formulation, administered orally as 5 capsules, matched for appearance, smell, taste, and weight. Those in the placebo arm received 5 placebo capsules, with the inert excipient being starch 1500. Participants were supervised 1:1 with a chaperone, who was supervised by a psychiatrist and the lead therapist. After 5–6 hours, the team assessed the participant for safety and discussed their experience with them. Participants were discharged 6–8 hours post-dosing.

Participants then attended follow-up visits at day 2, day 8, day 29, and day 85 after study drug administration.

The measures used in this analysis were as follows:

NEO-Five Factor Inventory (NEO-FFI; McCrae and Costa, 2004) at baseline, day 8, and day 85. The NEO-FFI measures five domains of personality: neuroticism, extroversion, openness, agreeableness, and conscientiousness.

Symptom Checklist-90 (SCL-90; Derogatis, 1994) at baseline, day 8, and day 85. The SCL-90 measures symptoms of psychopathology, with nine subscales: anger-hostility, anxiety, depression, interpersonal sensitivity, obsessive compulsive, paranoid ideation, phobic anxiety, psychoticism, and somatisation.

Life Changes Inventory (LCI; Greyson and Ring, 2004) at day 8 and day 85. The LCI measures changes in values and attitudes after near-death experiences, but has also been used to evaluate personal transformation following spiritually oriented experiences, including psychedelic experiences (Smigielski et al., 2019). Each item is rated on a five-point Likert scale ranging from −2 (strongly decreased) to +2 (strongly increased). Subscale scores (appreciation for life, self-acceptance, concern for others, concern with worldly achievement, concern with social/planetary values, quest for meaning/sense of purpose, spirituality, religiousness, and appreciation for death) and absolute change scores are calculated as the mean change of all items within that subscale or all 50 items, respectively. Participants were asked to indicate the change since the psilocybin experience.

Five-Dimensional Altered States of Consciousness Questionnaire (5D-ASC; Dittrich et al., 2006) at the study drug administration visit, 5–6 hours post-dosing. The 5D-ASC measures the acute drug effects across five dimensions: oceanic boundlessness, anxious ego dissolution, visual restructuralisation, auditory alterations, and vigilance reduction.

Intra-Extra Dimensional Set Shift (IED) task at day 8. The IED is a cognitive task that requires participants to learn a rule that determines the correct answer. After several responses, the stimuli and/or rule changes. The total error rate was used in this study to indicate difficulties with cognitive flexibility.

### Analysis

Participant baseline characteristics were summarised with descriptive statistics. The effects of psilocybin on personality (NEO-FFI) and psychiatric symptoms (SCL-90) at short- (day 8) and long-term follow-up (day 85) were analysed using a mixed-effects model for repeated measures (MMRM), with change from baseline score as the dependent variable. The model included fixed effects for visit, study drug, prior psilocybin experience, and study drug by visit interaction, with visit as the repeated factor, participant as a random effect, and baseline score as a covariate. An unstructured covariance matrix was used, and the Kenward and Roger method was used for calculating the denominator degrees of freedom for tests of fixed effects. The effects of psilocybin on values and beliefs (LCI) were analysed similarly; however, as this measure was only taken at day 8 and day 85 and not baseline (as it asks to what extent respondents felt their beliefs had changed since their experience), the LCI score was the dependent variable, and no covariates were entered into the model. Group differences were indicated where 95% confidence intervals did not include zero.

Since it was only taken at one time point post-dosing, a Kruskal–Wallis test was used to assess group differences in IED errors (due to a skewed distribution). If significant group differences were observed, this measure would have been taken forward in the following analyses as a proposed mediator, alongside 5D-ASC subscale scores.

Relationships between participant characteristics (age, sex, prior psilocybin experience), outcome measures (NEO-FFI, SCL-90, and LCI), and potential mediators were first explored with Spearman’s correlation analyses (or the point-biserial correlation for sex and past psychedelic experience). Given that significant correlations would be followed up with regression analyses, they were treated as exploratory and not corrected for multiple comparisons. Where significant correlations were found, variables were entered into hierarchical linear regressions to determine whether proposed mediators predicted change in outcome measures, whilst controlling for group and any associated demographic variables. Assumptions for hierarchical regressions were assessed with partial regression plots, and plots of studentised residuals against predicted values. Where plots indicated homoscedasticity, the dependent variable was square root transformed. Independence of residuals was assessed using the Durbin–Watson statistic and multicollinearity via variance inflation factor (VIF) values. Outliers with studentised deleted residuals greater than ±3 were excluded from analyses.

The SPSS macro PROCESS (Hayes, 2017) was used for mediation analyses. PROCESS uses a less conservative variant of the Sobel test, generating bias-corrected 95% bootstrap confidence intervals (CIs) for the indirect effect (based on 5000 samples).

Missing data on included measures ranged from 0.0% to 2.2% at baseline, 4.5% to 10.1% at day 8, and 10.1% to 14.6% at day 85. Because MMRM analyses all cases, imputation of missing values was not performed.

Analyses were conducted using SPSS V28 (Armonk, NY).

## Results

### Demographic characteristics

Baseline demographic participants by treatment group are displayed in Table 1.

**Table 1.** Baseline demographic characteristics.

Characteristic	Psilocybin 25 mg (*N* = 30)	Psilocybin 10 mg (*N* = 30)	Placebo (*N* = 29)
Gender, *n* (%)			
Male	16 (53.3)	16 (53.3)	16 (55.2)
Female	14 (46.7)	14 (46.7)	13 (44.8)
Race, *n* (%)			
White	25 (83.3)	27 (90.0)	20 (69.0)
Black	0	0	1 (3.4)
Asian	2 (6.7)	1 (3.3)	3 (10.3)
Mixed	2 (6.7)	1 (3.3)	1 (3.4)
Other	1 (3.3)	1 (3.3)	4 (13.8)
Age, mean (SD)	36.6 (10.29)	36.1 (9.25)	35.6 (7.69)
BMI, mean (SD)	22.9 (3.75)	23.0 (2.90)	23.7 (3.20)
Educational level, *n* (%)			
No formal qualifications	0	0	0
GCSE/GCE/O level	0	0	0
A level/NVQ	2 (6.7)	1 (3.3)	0
Undergraduate/Higher National Diploma	9 (30.0)	11 (36.7)	10 (34.5)
Masters or Postgraduate Diploma	16 (53.3)	16 (53.3)	15 (51.7)
PhD	3 (10.0)	2 (6.7)	4 (13.8)
Prior psilocybin experience, *n* (%)			
Yes	11 (36.7)	15 (50.0)	7 (24.1)
No	19 (63.3)	15 (50.0)	22 (75.9)

### Effects of psilocybin on personality, psychiatric symptoms, values, and cognitive flexibility

Table 2 presents the results from the mixed-effects models of change from baseline score on each subscale of the NEO-FFI, SCL-90, and LCI. The placebo and 10 mg psilocybin groups showed a significant decrease in NEO-FFI conscientiousness scores at day 85, and the 10 mg psilocybin group showed an increase in neuroticism scores at day 85. However, there were no meaningful differences in scores when comparing psilocybin groups to placebo at day 8 or 85. Regarding the SCL-90, obsessive-compulsive scores significantly decreased at day 8 in the placebo group, but there were no significant differences in scores when comparing the psilocybin groups to the placebo at day 8 or 85.

**Table 2.** Mixed model analyses of NEO-FFI, SCL-90, and LCI change scores.

	LS mean (SE) change from baseline in score		LS mean (95% CI) difference from placebo	
Variable	Day 8	Day 85	Day 8	Day 85
NEO-FFI agreeableness				
Placebo	0.23 (0.53)	−0.19 (0.64)	–	–
Psilocybin 10 mg	−0.22 (0.49)	−0.94 (0.58)	−0.45 (−1.89, 0.99)	−0.75 (−2.47, 0.97)
Psilocybin 25 mg	0.14 (0.50)	−0.86 (0.59)	−0.09 (−1.50, 1.31)	−0.67 (−2.37, 1.03)
NEO-FFI conscientiousness				
Placebo	−0.59 (0.60)	−1.51 (0.68)^a^	–	–
Psilocybin 10 mg	−0.14 (0.57)	−1.26 (0.61)^a^	0.45 (−1.20, 2.10)	0.25 (−1.56, 2.05)
Psilocybin 25 mg	0.12 (0.57)	−0.10 (0.62)	0.71 (−0.91, 2.34)	1.40 (−0.39, 3.20)
NEO-FFI extraversion				
Placebo	−0.13 (0.62)	−0.24 (0.77)	–	–
Psilocybin 10 mg	0.26 (0.57)	−0.89 (0.69)	0.39 (−1.28, 2.06)	−0.65 (−2.70, 1.39)
Psilocybin 25 mg	0.32 (0.58)	−0.72 (0.71)	0.45 (−1.19, 2.10)	−0.48 (−2.52, 1.55)
NEO-FFI neuroticism				
Placebo	0.36 (0.79)	1.76 (0.96)	–	–
Psilocybin 10 mg	0.19 (0.72)	2.06 (0.85)^a^	−0.17 (−2.28, 1.93)	0.30 (−2.23, 2.83)
Psilocybin 25 mg	0.19 (0.74)	0.78 (0.88)	−0.17 (−2.31, 1.96)	−0.98 (−3.55, 1.59)
NEO-FFI openness				
Placebo	−0.81 (0.55)	−0.71 (0.64)	–	–
Psilocybin 10 mg	0.30 (0.51)	0.39 (0.57)	1.11 (−0.37, 2.59)	1.10 (−0.60, 2.79)
Psilocybin 25 mg	0.41 (0.53)	−0.28 (0.59)	1.23 (−0.25, 2.71)	0.43 (−1.28, 2.13)
SCL-90 anger hostility				
Placebo	0.00 (0.03)	0.04 (0.05)	–	–
Psilocybin 10 mg	−0.05 (0.03)	0.02 (0.04)	−0.05 (−0.13, 0.03)	−0.02 (−0.14, 0.11)
Psilocybin 25 mg	−0.01 (0.03)	0.00 (0.05)	−0.01 (−0.09, 0.07)	−0.04 (−0.16, 0.09)
SCL-90 anxiety				
Placebo	−0.01 (0.04)	0.03 (0.07)	–	–
Psilocybin 10 mg	−0.03 (0.04)	−0.03 (0.06)	−0.02 (−0.14, 0.10)	−0.05 (−0.23, 0.13)
Psilocybin 25 mg	−0.02 (0.04)	−0.02 (0.06)	−0.01 (−0.12, 0.11)	−0.05 (−0.23, 0.13)
SCL-90 depression				
Placebo	0.01 (0.07)	0.02 (0.09)	–	–
Psilocybin 10 mg	0.04 (0.07)	0.06 (0.08)	0.03 (−0.17, 0.22)	0.04 (−0.19, 0.27)
Psilocybin 25 mg	0.03 (0.07)	0.06 (0.08)	0.02 (−0.17, 0.21)	0.05 (−0.18, 0.28)
SCL-90 interpersonal sensitivity				
Placebo	0.00 (0.05)	0.06 (0.06)	–	–
Psilocybin 10 mg	−0.04 (0.04)	0.00 (0.06)	−0.04 (−0.17, 0.09)	−0.06 (−0.23, 0.11)
Psilocybin 25 mg	0.02 (0.05)	0.02 (0.06)	0.02 (−0.11, 0.15)	−0.04 (−0.21, 0.13)
SCL-90 obsessive compulsive				
Placebo	−0.16 (0.07)^a^	−0.11 (0.07)	–	–
Psilocybin 10 mg	−0.06 (0.06)	−0.02 (0.06)	0.10 (−0.09, 0.29)	0.09 (−0.11, 0.28)
Psilocybin 25 mg	−0.06 (0.07)	−0.08 (0.07)	0.10 (−0.09, 0.28)	0.02 (−0.17, 0.21)
SCL-90 paranoid ideation				
Placebo	−0.05 (0.5)	−0.01 (0.4)	–	–
Psilocybin 10 mg	−0.01 (0.04)	0.05 (0.04)	0.04 (−0.09, 0.17)	0.06 (−0.05, 0.18)
Psilocybin 25 mg	0.06 (0.05)	0.06 (0.04)	0.11 (−0.02, 0.23)	0.07 (−0.05, 0.18)
SCL-90 phobic anxiety				
Placebo	0.00 (0.01)	0.00 (0.02)	–	–
Psilocybin 10 mg	−0.01 (0.01)	0.00 (0.02)	−0.01 (−0.05, 0.03)	−0.00 (−0.06, 0.05)
Psilocybin 25 mg	0.01 (0.01)	0.01 (0.02)	0.01 (−0.03, 0.04)	0.01 (−0.05, 0.07)
SCL-90 psychoticism				
Placebo	0.01 (0.03)	0.03 (0.03)	–	–
Psilocybin 10 mg	0.01 (0.03)	0.00 (0.03)	0.00 (−0.07, 0.08)	−0.03 (−0.11, 0.04)
Psilocybin 25 mg	0.02 (0.03)	0.03 (0.03)	0.01 (−0.07, 0.08)	−0.01 (−0.09, 0.07)
SCL-90 somatisation				
Placebo	0.00 (0.04)	0.00 (0.05)	–	–
Psilocybin 10 mg	0.06 (0.04)	0.01 (0.05)	0.06 (−0.06, 0.18)	0.01 (−0.13, 0.15)
Psilocybin 25 mg	0.06 (0.04)	0.04 (0.05)	0.06 (−0.06, 0.17)	0.04 (−0.10, 0.18)
LCI absolute change				
Placebo	0.11 (0.07)	0.10 (0.07)	–	–
Psilocybin 10 mg	0.48 (0.06)^a^	0.49 (0.06)^a^	0.37 (0.20, 0.55)^a^	0.39 (0.21, 0.57)^a^
Psilocybin 25 mg	0.42 (0.06)^a^	0.47 (0.06)^a^	0.31 (0.14, 0.48)^a^	0.37 (0.19, 0.55)^a^
LCI appreciation of life				
Placebo	0.18 (0.11)	0.16 (0.11)	–	–
Psilocybin 10 mg	0.78 (0.10)^a^	0.78 (0.09)^a^	0.61 (0.31, 0.90)^a^	0.63 (0.35, 0.90)^a^
Psilocybin 25 mg	0.63 (0.10)^a^	0.67 (0.10)^a^	0.45 (0.16, 0.74)^a^	0.52 (0.24, 0.80)^a^
LCI appreciation of death				
Placebo	0.05 (0.06)	0.07 (0.09)	–	–
Psilocybin 10 mg	0.17 (0.05)^a^	0.24 (0.07)^a^	0.12 (−0.04, 0.28)	0.18 (−0.05, 0.40)
Psilocybin 25 mg	0.14 (0.05)^a^	0.29 (0.08)^a^	0.10 (−0.06, 0.25)	0.22 (−0.01, 0.45)
LCI is concerned for others				
Placebo	0.15 (0.11)	0.15 (0.10)	–	–
Psilocybin 10 mg	0.54 (0.10)^a^	0.53 (0.08)^a^	0.39 (0.11, 0.67)^a^	0.38 (0.12, 0.64)^a^
Psilocybin 25 mg	0.57 (0.10)^a^	0.66 (0.09)^a^	0.43 (0.15, 0.71)^a^	0.51 (0.25, 0.77)^a^
LCI is concerned with social/planetary values				
Placebo	0.07 (0.06)	0.12 (0.08)	–	–
Psilocybin 10 mg	0.18 (0.05)^a^	0.24 (0.06)^a^	0.11 (−0.06, 0.27)	0.13 (−0.07, 0.32)
Psilocybin 25 mg	0.24 (0.06)^a^	0.25 (0.07)^a^	0.17 (0.01, 0.33)^a^	0.13 (−0.06, 0.32)
LCI is concerned with worldly achievement				
Placebo	−0.02 (0.06)	0.01 (0.07)	–	–
Psilocybin 10 mg	−0.22 (0.06)^a^	−0.24 (0.06)^a^	−0.20 (−0.37, −0.03)^a^	−0.25 (−0.44, −0.06)^a^
Psilocybin 25 mg	−0.16 (0.06)^a^	−0.09 (0.07)	−0.15 (−0.31, 0.02)	−0.10 (−0.29, 0.09)
LCI quest for meaning/sense of purpose				
Placebo	0.13 (0.09)	0.12 (0.11)	–	–
Psilocybin 10 mg	0.53 (0.08)^a^	0.51 (0.09)^a^	0.40 (0.15, 0.64)^a^	0.39 (0.11, 0.66)^a^
Psilocybin 25 mg	0.53 (0.09)^a^	0.47 (0.10)^a^	0.40 (0.16, 0.64)^a^	0.35 (0.07, 0.63)^a^
LCI religiousness				
Placebo	0.01 (0.05)	0.03 (0.07)	–	–
Psilocybin 10 mg	−0.04 (0.04)	−0.04 (0.07)	−0.05 (−0.17, 0.07)	−0.04 (−0.25, 0.17)
Psilocybin 25 mg	0.03 (0.04)	−0.00 (0.08)	0.03 (−0.10, 0.15)	0.03 (−0.19, 0.24)
LCI self-acceptance				
Placebo	0.09 (0.08)	0.10 (0.09)	–	–
Psilocybin 10 mg	0.76 (0.07)^a^	0.61 (0.08)^a^	0.67 (0.45, 0.89)^a^	0.51 (0.27, 0.75)^a^
Psilocybin 25 mg	0.62 (0.08)^a^	0.62 (0.08)^a^	0.53 (0.32, 0.75)^a^	0.52 (0.28, 0.76)^a^
LCI spirituality				
Placebo	0.19 (0.10)	0.13 (0.11)	–	–
Psilocybin 10 mg	0.51 (0.09)^a^	0.53 (0.09)^a^	0.32 (0.05, 0.59)^a^	0.40 (0.12, 0.68)^a^
Psilocybin 25 mg	0.43 (0.09)^a^	0.50 (0.10)^a^	0.24 (−0.03, 0.51)	0.37 (0.09, 0.65)^a^

CI: confidence interval; LCI: Life Changes Inventory; LS: least squares; NEO-FFI: NEO-Five Factor Inventory; SCL-90: Symptom Checklist-90; SE: standard error.

a 95% CIs did not include zero, indicating a statistically significant difference.

Regarding the LCI, there were no significant changes in absolute or subscale scores at day 8 or day 85 in the placebo group. Absolute change scores, appreciation for life, appreciation for death, concern for others, concern for social/planetary values, quest for meaning/sense of purpose, self-acceptance, and spirituality significantly increased in the 10 and 25 mg psilocybin groups at day 8 and day 85. Concern with worldly achievement significantly decreased in the 10 mg psilocybin group at day 8 and day 85, and in the 25 mg psilocybin group at day 8 only. Compared to placebo, absolute change scores, appreciation for life, concern for others, quest for meaning/sense of purpose, and self-acceptance were significantly increased in the 10 and 25 mg psilocybin groups at both day 8 and day 85. Compared to placebo, spirituality scores were significantly increased in the 10 mg group at day 8 and day 85, and in the 25 mg group at day 85. Compared to placebo, concern with social/planetary values was significantly increased at day 8 in the 25 mg psilocybin group only. Compared to the placebo, concern with worldly achievement was significantly decreased in the 10 mg psilocybin group at day 8 and day 85. There were no significant differences between the psilocybin groups on any measure at either time point.

There was no statistically significant difference in IED total errors between participants in the 25 mg psilocybin (median = 10.5, IQR = 6.3), 10 mg psilocybin (median = 11, IQR = 8.5), or placebo arms (median = 11, IQR = 9.5) at day 8, *H*(2) = 1.37, *p* = 0.505.

### Associations between outcome measures, participant characteristics, and proposed mediators

As there was no evidence of group differences in the SCL-90 and NEO-FFI, only the LCI outcome was explored further. Similarly, as there were no group differences in total IED errors, this variable was not considered further as a mediator. As there were no differences in LCI scores between the psilocybin groups, these groups were combined for correlation analyses. Correlations between age, sex, prior psilocybin use, proposed mediators (5D-ASC subscales), and LCI subscales in the 10 and 25 mg psilocybin groups are shown in Table 3. Several 5D-ASC subscales were significantly associated with LCI change scores. There were no significant correlations between any variables in the placebo group.

**Table 3.** Correlations between LCI domains, demographic characteristics, and proposed mediators in 10 and 25 mg psilocybin dosing groups (*n* = 60).

	Age		Sex		Prior psilocybin use		5D-ASC anxious ego dissolution		5D-ASC auditory alterations		5D-ASC oceanic boundlessness		5D-ASC vigilance reduction		5D-ASC visual restructuralisation	
Variable	ρ	*p*	*r*_pb_	*p*	*r*_pb_	*p*	ρ	*p*	ρ	*p*	ρ	*p*	ρ	*p*	ρ	*p*
LCI absolute change day 8	0.142	0.283	−0.068	0.606	0.062	0.643	−0.036	0.789	0.299*	0.021	0.511*	0.000	−0.115	0.386	0.295*	0.023
LCI absolute change day 85	0.220	0.100	−0.048	0.725	−0.083	0.537	−0.206	0.125	0.069	0.611	0.307*	0.020	−0.087	0.519	0.115	0.393
LCI appreciation for life day 8	0.147	0.266	0.096	0.471	0.028	0.833	−0.027	0.841	0.297*	0.022	0.449*	0.000	−0.102	0.440	0.188	0.154
LCI appreciation for life day 85	0.182	0.176	0.052	0.701	−0.124	0.360	−0.157	0.244	0.199	0.137	0.341*	0.010	−0.100	0.460	0.152	0.258
LCI appreciation for death day 8	0.060	0.653	−0.198	0.133	0.122	0.356	−0.162	0.219	0.132	0.320	0.449*	<0.001	−0.105	0.428	0.272*	0.037
LCI appreciation for death day 85	0.391*	0.003	−0.003	0.983	−0.087	0.519	−0.084	0.537	0.027	0.841	0.271*	0.041	−0.159	0.237	0.120	0.375
LCI is concerned for others day 8	0.272*	0.037	−0.100	0.453	0.115	0.388	−0.229	0.081	0.198	0.133	0.450*	0.000	−0.243	0.064	0.251	0.055
LCI is concerned for others day 85	0.317*	0.016	−0.141	0.294	−0.046	0.734	−0.205	0.125	0.106	0.435	0.281*	0.034	−0.031	0.820	0.105	0.437
LCI is concerned with social/planetary values day 8	0.030	0.824	0.256	0.050	−0.272*	0.038	−0.004	0.975	0.277*	0.034	0.190	0.149	−0.024	0.859	0.184	0.164
LCI is concerned with social/planetary values day 85	0.014	0.915	−0.007	0.956	0.061	0.653	−0.219	0.102	0.173	0.198	0.065	0.633	−0.218	0.103	0.118	0.381
LCI is concerned with worldly achievement day 8	−0.039	0.768	−0.075	0.573	0.113	0.392	−0.107	0.421	−0.308*	0.018	−0.336*	0.009	0.008	0.952	−0.221	0.092
LCI is concerned with worldly achievement day 85	−0.303*	0.022	−0.027	0.845	0.085	0.531	0.143	0.288	0.056	0.679	−0.111	0.413	0.304*	0.022	0.069	0.612
LCI quest for meaning/sense of purpose day 8	0.148	0.262	−0.100	0.450	0.060	0.652	−0.003	0.984	0.271*	0.038	0.511*	0.000	−0.029	0.827	0.304*	0.019
LCI quest for meaning/sense of purpose day 85	0.235	0.079	0.152	0.259	−0.023	0.866	−0.133	0.323	0.237	0.076	0.543*	0.000	−0.006	0.963	0.304*	0.022
LCI religiousness day 8	0.048	0.716	0.083	0.534	0.048	0.718	−0.054	0.686	0.001	0.993	0.144	0.277	0.010	0.940	0.289*	0.026
LCI religiousness day 85	0.081	0.548	−0.149	0.270	−0.084	0.534	0.104	0.440	0.090	0.503	0.081	0.549	0.245	0.066	0.243	0.069
LCI self-acceptance day 8	0.190	0.150	0.028	0.832	0.085	0.520	−0.057	0.670	0.241	0.066	0.373*	0.004	−0.125	0.344	0.245	0.062
LCI self-acceptance day 85	0.178	0.186	−0.021	0.877	−0.066	0.623	−0.170	0.205	−0.031	0.816	0.276*	0.038	0.050	0.713	0.064	0.634
LCI spirituality day 8	0.078	0.555	−0.053	0.690	0.136	0.303	−0.081	0.542	0.132	0.320	0.461*	0.000	−0.250	0.056	0.221	0.092
LCI spirituality day 85	0.101	0.453	−0.056	0.681	−0.042	0.755	−0.245	0.066	−0.029	0.831	0.264*	0.047	−0.085	0.531	0.072	0.594

5D-ASC: Five-Dimensional Altered States of Consciousness Questionnaire; LCI: Life Changes Inventory; *p: p*-value; ρ: Spearman’s rho; *r*_pb_: point-biserial correlation.

* Correlation is significant at the 0.05 level (two-tailed).

#### LCI absolute change

As there were no group differences in change in religiousness or change in appreciation of death at either follow-up point, or concern with social/planetary values at day 85, these subscales of the LCI were excluded from further analyses. Given the association between LCI absolute change scores at day 8, 5D-ASC auditory alterations, 5D-ASC oceanic boundlessness, and 5D-ASC visual restructuralisation, a hierarchical regression was run to examine whether these aspects of the psychedelic experience predicted LCI absolute change, with group entered alone in the first model, and subscales entered in the second model (Table 4). The addition of the ASC subscales in the second model led to a significant increase in *R*^2^ (*p* < 0.001); however, of these, only oceanic boundlessness made a significant unique contribution to explaining the variance in LCI change scores. To explore a possible mediational effect of oceanic boundlessness, a mediation analysis was run with group as the independent variable (using indicator coding, with placebo as the reference group), 5D-ASC oceanic boundlessness score as the mediator, and LCI absolute change score at day 8 as the dependent variable. Bias-corrected bootstrapped CIs for the indirect effects were entirely above zero (*b*_1_ = 0.30 [0.16 − 0.46], *b*_2_ = 0.35 [0.19 − 0.53]), indicating a significant mediation effect of group on LCI absolute change at day 8 through oceanic boundlessness. The direct effect of group in the presence of the mediator was also significant (*c*_1_ = 0.27, *c*_2_ = 0.16, *p* = 0.007), indicating partial mediation.

**Table 4.** Hierarchical regression analyses predicting LCI absolute change score at day 8 and day 85 from associated 5D-ASC subscales.

Variable	Model 1	Model 2
LCI absolute change score at day 8		
Group		
10 mg vs placebo	0.80*	0.38*
25 mg vs placebo	0.71*	0.22
5D-ASC auditory alterations		0.11
5D-ASC oceanic boundlessness		0.50*
5D-ASC visual restructuralisation		−0.02
*R*^2^	0.48*	0.62*
LCI absolute change score at day 85		
Group		
10 mg vs placebo	0.73*	0.44*
25 mg vs placebo	0.66*	0.32
5D-ASC oceanic boundlessness		0.36*
*R*^2^	0.62*	0.66*

Figures shown are standardised coefficients. The group was represented as two dummy variables.

5D-ASC: Five-Dimensional Altered States of Consciousness Questionnaire; LCI: Life Changes Inventory.

* *p* < 0.05.

Given the association between LCI absolute change scores at day 85 and 5D-ASC oceanic boundlessness, a hierarchical regression was run to examine whether this subscale predicted LCI absolute change at day 85, with group entered alone in the first model, and oceanic boundlessness entered in the second model (Table 4). The addition of oceanic boundlessness in the second model led to a significant increase in *R*^2^ (*p* = 0.013) and made a significant unique contribution to explaining the variance in LCI change scores. To explore a possible mediational effect, a mediation analysis was run with group as the independent variable, 5D-ASC oceanic boundlessness score as the mediator, and LCI absolute change score at day 85 as the dependent variable. Bias-corrected bootstrapped CIs for the indirect effects were entirely above zero (*b*_1_ = 0.20 [0.05 – 0.37], *b*_2_ = 0.24 [0.06 – 0.44]), indicating a significant mediation effect of group on LCI absolute change at day 85 through oceanic boundlessness. The direct effect of group in the presence of the mediator was also significant (*c*_1_ = 0.31, *c*_2_ = 0.23, *p* = 0.020), indicating partial mediation.

#### LCI subscales

Analyses for each subscale are presented in the Supplemental Material and summarised here. 5D-ASC oceanic boundlessness fully mediated the effects of group on LCI appreciation for life at day 8 and day 85, concern for others at day 8 (but not at day 85), quest for meaning/sense of purpose at day 8 and day 85, and spirituality at day 8 (but not at day 85), and partially mediated the effects of group on LCI self-acceptance at day 8 (but not at day 85). 5D-ASC auditory alterations fully mediated LCI concern with social/planetary values day 8. Regression analyses for LCI concerned with worldly achievement at day 8 and day 85 were either not significant or suggested that predictors did not significantly explain variance in the model.

## Discussion

In this sample of 89 healthy volunteers randomised to receive a single dose of psilocybin 25 mg, 10 mg, or an inert placebo, we found significant changes in personal values in several domains of the LCI at both short-and longer-term follow-up in those who received psilocybin, compared to those who received a placebo. Specifically, both psilocybin groups showed an increase in LCI absolute change, appreciation for life, concern for others, quest for meaning/sense of purpose, and self-acceptance at short- and long-term follow-up, compared to placebo. Similarly, spirituality was increased in both psilocybin groups compared to placebo at long-term follow-up, and at short-term follow-up in the 10 mg group only. These changes were either partially or fully mediated by the oceanic boundlessness subscale of the 5D-ASC, although this mediation effect did not hold for longer-term follow-up for some of the LCI subscales. Concern with worldly achievement was reduced at short- and long-term follow-up in the 10 mg group compared to placebo, and concern with social/planetary values was increased in the 25 mg group at short-term follow-up only. Changes in the latter domain were fully mediated by the auditory alterations subscale of the 5D-ASC. There were no between-group differences in IED errors at day 8; therefore, cognitive flexibility was not tested as a possible mediator. No differences in personality, as measured by the NEO-FFI, or psychiatric symptoms, as measured by the SCL-90, were found between groups.

Although significant reductions in depression and anxiety have been demonstrated in trials testing psilocybin for the treatment of psychiatric disorders (Goldberg et al., 2020; Haikazian et al., 2023), we did not find any differences in psychiatric symptoms in our healthy volunteer sample. This is unsurprising, given that those with current or prior psychiatric disorders were excluded. Similarly, studies in participants with TRD have reported changes in neuroticism, extraversion, and openness following treatment with psilocybin, in the direction of normative scores (Erritzoe et al., 2018; Weiss et al., 2024). Studies in healthy volunteers have also found increases in openness following psilocybin administration (MacLean et al., 2011; Madsen et al., 2020), a finding that was not replicated in the present study. This might be due to a relatively high proportion of participants with prior psilocybin experience in the present study (37%), compared to exclusively naïve samples in past studies (MacLean et al., 2011; Madsen et al., 2020). Relatedly, mean openness scores at baseline in our trial population (mean = 34.4) were higher than published population norms (mean = 26.5; Egan et al., 2000), indicating participants in this trial were already more open to experience before receiving psilocybin. However, NEO-PI scores reported in previous healthy volunteer studies were also higher than population norms; therefore, differences in openness are unlikely to be the reason for conflicting results (MacLean et al., 2011; Madsen et al., 2020).

Our findings regarding changes in personal values are consistent with past research reporting increased altruism/concern for others, positive attitudes about oneself and/or life, appreciation of spirituality, and reduced materialism after psychedelic use in healthy volunteers (Griffiths et al., 2006, 2008, 2018; Lerner and Lyvers, 2006; Schmid and Liechti, 2018). The increases in self-acceptance and appreciation for life observed at both short- and long-term follow-up in this study may hold particular promise for the treatment of psychiatric disorders such as MDD, which are associated with a negative outlook and view of the self. Although few trials have examined changes in values with psilocybin treatment in patient samples, qualitative work in TRD has reported that patients experience psilocybin treatment as bringing a sense of connection to oneself and others and increasing self-worth (Watts et al., 2017). Almost half of these patients reported major lifestyle changes following treatment (e.g. diet, smoking, or alcohol use), indicating that changes in outlook may lead to behavioural change. Offering a possible mechanism for psilocybin’s therapeutic effects, it has been hypothesised that serotonergic psychedelics lead to a relaxation of neuronally encoded predictive models, which may manifest as a reduction in subjectively felt confidence of one’s beliefs, allowing for change (Carhart-Harris and Friston, 2019). However, empirical studies relating these concepts to brain activity during psilocybin administration are still required.

Oceanic boundlessness was found to mediate many of the changes in beliefs found in participants who received psilocybin, compared to those who received a placebo. Oceanic boundlessness refers to the derealisation and depersonalisation associated with positive emotional states, including euphoria, unitive states, and spiritual experiences. The closely related concept of mystical experiences has been found to predict variance in attitudes towards life, spirituality, and mood following psilocybin administration in healthy volunteers (Griffiths et al., 2018), and a recent meta-analysis in patient populations suggested an association between mystical experiences and improvements in symptoms (Ko et al., 2022). Interestingly, a previous study using LSD in healthy volunteers showed that the oceanic boundlessness subscale score of the 5D-ASC was significantly correlated with changes in well-being/life satisfaction and death transcendence at 12-month follow-up, whilst auditory alterations were significantly associated with positive mood changes, altruistic social effects, and personal meaning of the experience (Schmid and Liechti, 2018). In our study, auditory alterations were found to fully mediate differences in concern with social/planetary values at short-term follow-up between groups. It may be that auditory hallucinations associated with psilocybin and other classic psychedelics are perceived as subjectively meaningful, in a similar way to enhanced appreciation for music (Kaelen et al., 2018).

That cognitive flexibility, as measured by the IED, did not seem to differ based on group allocation is somewhat surprising, given its hypothesised role as a potential mechanism for the therapeutic effects of psilocybin (Torrado Pacheco et al., 2023). However, it must be noted that the IED task was only administered at day 8; baseline performance was not measured. This is a significant limitation of the trial design; examining change scores from baseline would have been a more valid approach. Relatedly, our findings regarding cognitive flexibility may be due to the use of a healthy control sample that likely did not have difficulties with cognitive flexibility before receiving psilocybin. Indeed, the distribution of IED errors at day 8 was positively skewed, possibly indicating a ceiling effect.

In clinical samples, psilocybin and other classic psychedelics may improve cognitive flexibility; however, evidence is mixed as to whether these improvements are associated with therapeutic outcomes. In those with depression, a past study did not find that improvements in cognitive flexibility were related to improvements in depression (Doss et al., 2021), whilst another study found strong associations between the two (Sloshower et al., 2024). This may be due to differences in the measurement of cognitive flexibility. In the former study, cognitive flexibility was measured using a performance-based measure, the Penn Conditional Exclusion Test. On the other hand, Sloshower and colleagues used the Acceptance and Action Questionnaire-II (AAQ-II), which may be more suitable for assessing psychological flexibility in relation to therapeutic interventions. Performance-based measures, such as the IED and the Penn Conditional Exclusion Test, involve a host of cognitive processes in addition to set-shifting, which may affect task performance. Future research in this area should include measures that are sensitive to psychological or cognitive flexibility as it pertains to psychopathology and well-being.

A number of strengths and limitations of the current work should be noted. Most studies examining personality, value changes, and cognitive flexibility with psilocybin or other classic psychedelics have used surveys or open-label one-arm designs (Doss et al., 2021; Erritzoe et al., 2018; Haijen et al., 2018; Murphy-Beiner and Soar, 2020; Schmid and Liechti, 2018). The addition of a placebo control group is a significant strength of the current study. However, given the pronounced subjective effects of psilocybin, it is very likely that functional unblinding occurred, whereby participants would have been aware of whether they received psilocybin or a placebo. A superior design may have been the addition of an active control, such as another non-psychedelic psychoactive drug or a very low dose of psilocybin (lower than the 10 mg dose used in this study, which would still produce pronounced subjective effects). Another limitation is the relatively small sample size, which may have resulted in inadequate power to detect significant group differences in our outcome measures. Conversely, the large number of statistical tests raises the possibility of Type I errors. This exploratory analysis should be replicated in larger RCTs.

Finally, two of the outcome measures used were self-report measures and may have been subject to recall biases, particularly the LCI, as it asks respondents to indicate changes in values since their experience. Due to functional unblinding, participants who received psilocybin may have reported more changes in values because of pre-existing beliefs about psilocybin and its effects. This may be particularly true for those who have prior experience with psilocybin, as was the case for a sizable proportion of this sample. It is also notable that no significant differences were found in Scale of Social Responsibility (Singhapakdi et al., 1996) and Toronto Empathy Questionnaire (Spreng et al., 2009) scores measured pre- and post-intervention between groups in the primary analysis of this trial (Rucker et al., 2022). These scales show some overlap with some subscales of the LCI, including those that did show change in this analysis, questioning the validity of the LCI as a measure of change. Relatedly, although the LCI is the most widely used instrument for quantifying changes in values following near-death experiences (Greyson and Ring, 2004) and changes in values have been shown to persist long-term (Greyson, 2022), it has rarely been used to assess values after psychedelic experiences and has not been validated in this context.

## Conclusions

This analysis suggests that aspects of the acute psychedelic experience, namely oceanic boundlessness and, to a lesser extent, auditory alterations, mediate changes in personal values in healthy volunteers administered a single dose of 10 or 25 mg psilocybin versus placebo. These findings suggest that acute feelings of euphoria, unitive states, and spiritual experiences are key to some of the changes in values reported after psilocybin use. These findings may have clinical applications, for example, in MDD, which is characterised by a negative view of the self, world, and future. Cognitive flexibility was not found to differ between groups, but this might be due to the suboptimal measurement in this trial and/or the low ecological validity of set-shifting tasks such as the IED. Our findings did not suggest a significant effect of a single dose of psilocybin on personality and psychiatric symptoms compared to placebo, but this study was not necessarily powered to detect such an effect.

## Supplemental Material

sj-docx-1-jop-10.1177_02698811251408769 – Supplemental material for Effects of psilocybin on personality, psychiatric symptoms, and values: Exploring mediating effects of the acute psychedelic experience Supplemental material, sj-docx-1-jop-10.1177_02698811251408769 for Effects of psilocybin on personality, psychiatric symptoms, and values: Exploring mediating effects of the acute psychedelic experience by Jess Kerr-Gaffney, Samuel Myrtle, Famia Askari, Catherine Bird, Nadav Liam Modlin, Allan H. Young and James Rucker in Journal of Psychopharmacology
