# Supplementary material for: Effects of psilocybin on personality, psychiatric symptoms, and values: Exploring mediating effects of the acute psychedelic experience
Source: J Psychopharmacol. 2026 Jan 26;40(5):806–17. doi: 10.1177/02698811251408769 (PMC13310281; doi:10.1177/02698811251408769)
Supplement: sj-docx-1-jop-10.1177_02698811251408769 – Supplemental material for Effects of psilocybin on personality, psychiatric symptoms, and values: Exploring mediating effects of the acute psychedelic experience [file sj-docx-1-jop-10.1177_02698811251408769.docx]

**Supplementary material: LCI subscale regression and mediation analyses**

**LCI Appreciation for Life at Day 8**

A hierarchical regression was run to examine whether associated 5D-ASC subscales (auditory alterations and oceanic boundlessness) predicted LCI appreciation for life at day 8, with group entered alone in the first model, and the subscales entered in the second model (Table S1). The addition of the 5D-ASC subscales in the second model led to a significant increase in R^2^ (p <.001), however, of these, only oceanic boundlessness made a significant unique contribution to explaining the variance in LCI change scores. To explore a possible mediational effect of oceanic boundlessness, a mediation analysis was run with group as the independent variable (using indicator coding, with placebo as the reference group), 5D-ASC oceanic boundlessness score as the mediator, and LCI appreciation for life score at day 8 as the dependent variable. Bias corrected bootstrapped CIs for the indirect effects were entirely above zero (b_1_ = 0.54 [0.26 – 0.86], b_2_ = 0.62 [0.31 – 0.94]), indicating a significant mediation effect of group on LCI appreciation for life at day 8 through oceanic boundlessness. The direct effect of group in the presence of the mediator was not significant (c_1_ = 0.18, c_2_ = -0.04, p = .122), indicating full mediation.

| Table S1. Hierarchical regression analysis predicting LCI appreciation for life score at day 8 from associated 5D-ASC subscales | | |
| --- | --- | --- |
|  | Model 1 | Model 2 |
| Group |  |  |
| 10mg vs placebo | 0.63* | 0.16 |
| 25mg vs placebo | 0.50* | -0.06 |
| 5D-ASC auditory alterations |  | 0.11 |
| 5D-ASC oceanic boundlessness |  | 0.55* |
| R^2^ | 0.28* | 0.44* |
| Figures shown are standardised coefficients. Group was represented as two dummy variables. * *p* <.05 | | |

**LCI Appreciation for Life at Day 85**

A hierarchical regression was run to examine whether 5D-ASC oceanic boundlessness predicted LCI appreciation for life at day 85, with group entered alone in the first model, and the subscales entered in the second model (Table S2). The addition of the oceanic boundlessness in the second model led to a significant increase in R^2^ (p = .003) and made a significant unique contribution to explaining the variance in LCI change scores. To explore a possible mediational effect of oceanic boundlessness, a mediation analysis was run with group as the independent variable, 5D-ASC oceanic boundlessness score as the mediator, and LCI appreciation for life score at day 85 as the dependent variable. Bias corrected bootstrapped CIs for the indirect effects were entirely above zero (b_1_ = 0.40 [0.12 – 0.70], b_2_ = 0.48 [0.15 – 0.82]), indicating a significant mediation effect of group on LCI appreciation for life at day 85 through oceanic boundlessness. The direct effect of group in the presence of the mediator was not significant (c_1_ = 0.28, c_2_ = 0.14, p = .200), indicating full mediation.

| Table S2. Hierarchical regression analysis predicting LCI appreciation for life score at day 85 from associated 5D-ASC subscales | | |
| --- | --- | --- |
|  | Model 1 | Model 2 |
| Group |  |  |
| 10mg vs placebo | 0.63* | 0.26 |
| 25mg vs placebo | 0.55* | 0.12 |
| 5D-ASC oceanic boundlessness |  | 0.46* |
| R^2^ | 0.27* | 0.36* |
| Figures shown are standardised coefficients. Group was represented as two dummy variables. * *p* <.05 | | |

**LCI Concern for Others at Day 8**

A hierarchical regression was run to examine whether 5D-ASC oceanic boundlessness predicted LCI concern for others at day 8, with group and age in the first model, and oceanic boundlessness added in the second model (Table S3). The addition of oceanic boundlessness in the second model led to a significant increase in R^2^ (p < .001) and made a significant unique contribution to explaining the variance in LCI scores. Age was not significant in either model, and group became non-significant in the second model. To explore a possible mediational effect of oceanic boundlessness, a mediation analysis was run with group as the independent variable, 5D-ASC oceanic boundlessness score as the mediator, and LCI concern for others at day 8 as the dependent variable. Bias corrected bootstrapped CIs for the indirect effects were entirely above zero (b_1_ = 0.52 [0.25 – 0.84], b_2_ = 0.59 [0.29 – 0.93]), indicating a significant mediation effect of group on LCI concern for others at day 8 through oceanic boundlessness. The direct effect of group in the presence of the mediator was not significant (c_1_ = -0.04, c_2_ = -0.08, p = .899), indicating full mediation.

| Table S3. Hierarchical regression analysis predicting LCI concern for others score at day 8 from associated 5D-ASC subscales and demographic variables | | |
| --- | --- | --- |
|  | Model 1 | Model 2 |
| Group |  |  |
| 10mg vs placebo | 0.43* | -0.02 |
| 25mg vs placebo | 0.45* | -0.07 |
| Age | 0.20 | 0.18 |
| 5D-ASC oceanic boundlessness |  | 0.59* |
| R^2^ | 0.27* | 0.36* |
| Figures shown are standardised coefficients. Group was represented as two dummy variables. * *p* <.05 | | |

**LCI Concern for Others at Day 85**

A hierarchical regression was run to examine whether 5D-ASC oceanic boundlessness predicted LCI concern for others at day 85, with group and age in the first model, and oceanic boundlessness added in the second model (Table S4). The addition of oceanic boundlessness in the second model led to a significant increase in R^2^ (p = .030) and made a significant unique contribution to explaining the variance in LCI scores. Age was not significant in either model, and group became non-significant in the second model. To explore a possible mediational effect of oceanic boundlessness, a mediation analysis was run with group as the independent variable, 5D-ASC oceanic boundlessness score as the mediator, and LCI concern for others at day 85 as the dependent variable. Bias corrected bootstrapped CIs for the indirect effects included zero (b_1_ = 0.30 [-0.01 – 0.61], b_2_ = 0.35 [-0.01 – 0.73]), indicating there was no significant mediation effect of group on LCI concern for others at day 85 through oceanic boundlessness.

| Table S4. Hierarchical regression analysis predicting LCI concern for others score at day 85 from associated 5D-ASC subscales and demographic variables | | |
| --- | --- | --- |
|  | Model 1 | Model 2 |
| Group |  |  |
| 10mg vs placebo | 0.39* | 0.10 |
| 25mg vs placebo | 0.50* | 0.16 |
| Age | 0.16 | 0.16 |
| 5D-ASC oceanic boundlessness |  | 0.36* |
| R^2^ | 0.20* | 0.26* |
| Figures shown are standardised coefficients. Group was represented as two dummy variables. * *p* <.05 | | |

**Concern with Social / Planetary Values Day 8**

A hierarchical regression was run to examine whether 5D-ASC auditory alterations predicted LCI concern with social / planetary values at day 8, with group and prior psilocybin experience in the first model, and auditory alterations added in the second model (Table S5). The addition of auditory alterations in the second model led to a significant increase in R^2^ (p = .004) and made a significant unique contribution to explaining the variance in LCI scores. Prior psilocybin experience was not significant in either model, and group became non-significant in the second model. To explore a possible mediational effect of auditory alterations, a mediation analysis was run with group as the independent variable, 5D-ASC auditory alterations score as the mediator, and LCI concern with social/ planetary values at day 8 as the dependent variable. Bias corrected bootstrapped CIs for the indirect effects were entirely above zero (b_1_ = 0.13 [0.04 – 0.23], b_2_ = 0.21 [0.07 – 0.35]), indicating a significant mediation effect of group on LCI concern with social/ planetary values at day 8 through auditory alterations. The direct effect of group in the presence of the mediator was not significant (c_1_ = 0.11, c_2_ = 0.05, p = .539), indicating full mediation.

| Table S5. Hierarchical regression analysis predicting LCI Concern with Social/ Planetary Values Day 8 from associated 5D-ASC subscales and demographic variables | | |
| --- | --- | --- |
|  | Model 1 | Model 2 |
| Group |  |  |
| 10mg vs placebo | 0.35* | 0.17 |
| 25mg vs placebo | 0.36* | 0.08 |
| Prior psilocybin experience | -0.15 | -0.11 |
| 5D-ASC auditory alterations |  | 0.38* |
| R^2^ | 0.13* | 0.22* |
| Figures shown are standardised coefficients. Group was represented as two dummy variables. * *p* <.05 | | |

**LCI Concern with Worldly Achievement Day 8**

A hierarchical regression was run to examine whether 5D-ASC auditory alterations and oceanic boundlessness predicted LCI concern with worldly achievement at day 8, with group in the first model, and the LCI subscales added in the second model (Table S6). Neither model was significant, therefore no mediation analysis was run.

| Table S6. Hierarchical regression analysis predicting Concern with Worldly Achievement Day 8 from associated 5D-ASC subscales. | | |
| --- | --- | --- |
|  | Model 1 | Model 2 |
| Group |  |  |
| 10mg vs placebo | -0.24 | -0.02 |
| 25mg vs placebo | -0.22 | 0.05 |
| 5D-ASC oceanic boundlessness |  | -0.05 |
| 5D-ASC auditory alterations |  | -0.28 |
| R^2^ | 0.02 | 0.04 |
| Figures shown are standardised coefficients. Group was represented as two dummy variables. * *p* <.05 | | |

**LCI Concern with Worldly Achievement Day 85**

A hierarchical regression was run to examine whether 5D-ASC visual restructuralization predicted LCI concern with worldly achievement at day 85, with group and age in the first model, and visual restructuralization added in the second model (Table S7). The addition of visual restructuralization in the second model did not significantly increase R^2^ (p = .886) and did not significantly contribute to explaining the variance in LCI scores in either model, therefore a mediation analysis was not run.

| Table S7. Hierarchical regression analysis predicting LCI Concern with Worldly achievement at Day 85 from associated 5D-ASC subscales and demographic variables | | |
| --- | --- | --- |
|  | Model 1 | Model 2 |
| Group |  |  |
| 10mg vs placebo | -0.31* | -0.34 |
| 25mg vs placebo | -0.02 | -0.05 |
| Age | -0.31* | -0.31* |
| 5D-ASC visual restructuralization |  | 0.03 |
| R^2^ | 0.13* | 0.22* |
| Figures shown are standardised coefficients. Group was represented as two dummy variables. * *p* <.05 | | |

**LCI Quest for Meaning/Sense of Purpose Day 8**

A hierarchical regression was run to examine whether 5D-ASC auditory alterations, oceanic boundlessness, and visual restructuralization predicted LCI quest for meaning/sense of purpose at day 8, with group in the first model, and the 5D-ASC subscales added in the second model (Table S8). The addition of the subscales in the second model led to a significant increase in R^2^ (p < .001), however only oceanic boundlessness made a significant unique contribution to explaining the variance in LCI scores. To explore a possible mediational effect of oceanic boundlessness, a mediation analysis was run with group as the independent variable, 5D-ASC oceanic boundlessness as the mediator, and LCI quest for meaning/sense of purpose at day 8 as the dependent variable. Bias corrected bootstrapped CIs for the indirect effects were entirely above zero (b_1_ = 0.54 [0.28 – 0.85], b_2_ = 0.62 [0.34 – 0.92]), indicating a significant mediation effect of group on LCI quest for meaning/sense of purpose at day 8 through oceanic boundlessness. The direct effect of group in the presence of the mediator was not significant (c_1_ = -0.12, c_2_ = -0.20, p = .465), indicating full mediation.

| Table S8. Hierarchical regression analysis predicting LCI Quest for Meaning/Sense of Purpose at Day 8 from associated 5D-ASC subscales | | |
| --- | --- | --- |
|  | Model 1 | Model 2 |
| Group |  |  |
| 10mg vs placebo | 0.42* | -0.07 |
| 25mg vs placebo | 0.42* | -0.13 |
| 5D-ASC auditory alterations |  | -0.06 |
| 5D-ASC oceanic boundlessness |  | 0.81* |
| 5D-ASC Visual Restructuralization |  | -0.12 |
| R^2^ | 0.15* | 0.37* |
| Figures shown are standardised coefficients. Group was represented as two dummy variables. * *p* <.05 | | |

**LCI Quest for Meaning/Sense of Purpose Day 85**

A hierarchical regression was run to examine whether 5D-ASC oceanic boundlessness and visual restructuralization predicted LCI quest for meaning/sense of purpose at day 85, with group in the first model, and the 5D-ASC subscales added in the second model (Table S9). The addition of the subscales in the second model led to a significant increase in R^2^ (p < .001), however only oceanic boundlessness made a significant unique contribution to explaining the variance in LCI scores. To explore a possible mediational effect of oceanic boundlessness, a mediation analysis was run with group as the independent variable, 5D-ASC oceanic boundlessness as the mediator, and LCI quest for meaning/sense of purpose at day 85 as the dependent variable. Bias corrected bootstrapped CIs for the indirect effects were entirely above zero (b_1_ = 0.64 [0.33 – 1.01], b_2_ = 0.76 [0.42 – 1.16]), indicating a significant mediation effect of group on LCI quest for meaning/sense of purpose at day 85 through oceanic boundlessness. The direct effect of group in the presence of the mediator was not significant (c_1_ = -0.20, c_2_ = -0.33, p = .243), indicating full mediation.

| Table S9. Hierarchical regression analysis predicting LCI Quest for Meaning/Sense of Purpose at Day 85 from associated 5D-ASC subscales | | |
| --- | --- | --- |
|  | Model 1 | Model 2 |
| Group |  |  |
| 10mg vs placebo | 0.42* | -0.08 |
| 25mg vs placebo | 0.40* | -0.19 |
| 5D-ASC oceanic boundlessness |  | 0.88* |
| 5D-ASC Visual Restructuralization |  | -0.23 |
| R^2^ | 0.13* | 0.37* |
| Figures shown are standardised coefficients. Group was represented as two dummy variables. * *p* <.05 | | |

**LCI Self-acceptance Day 8**

A hierarchical regression was run to examine whether 5D-ASC oceanic boundlessness predicted LCI self-acceptance at day 8, with group in the first model, and 5D-ASC oceanic boundlessness added in the second model (Table S10). The addition of oceanic boundlessness in the second model led to a significant increase in R^2^ (p < .001). To explore a possible mediational effect of oceanic boundlessness, a mediation analysis was run with group as the independent variable, 5D-ASC oceanic boundlessness as the mediator, and LCI self-acceptance at day 8 as the dependent variable. Bias corrected bootstrapped CIs for the indirect effects were entirely above zero (b_1_ = 0.35 [0.13 – 0.60], b_2_ = 0.40 [0.16 – 0.69]), indicating a significant mediation effect of group on LCI self-acceptance at day 8 through oceanic boundlessness. The direct effect of group in the presence of the mediator was significant (c_1_ = 0.32, c_2_ = 0.13, p = .037), indicating partial mediation.

| Table S10. Hierarchical regression analysis predicting LCI Self-Acceptance at day 8 from associated 5D-ASC subscales | | |
| --- | --- | --- |
|  | Model 1 | Model 2 |
| Group |  |  |
| 10mg vs placebo | 0.68* | 0.32* |
| 25mg vs placebo | 0.55* | 0.13 |
| 5D-ASC oceanic boundlessness |  | 0.47* |
| R^2^ | 0.32* | 0.40* |
| Figures shown are standardised coefficients. Group was represented as two dummy variables. * *p* <.05 | | |

**LCI Self-acceptance Day 85**

A hierarchical regression was run to examine whether 5D-ASC oceanic boundlessness predicted LCI self-acceptance at day 85, with group in the first model, and 5D-ASC oceanic boundlessness added in the second model (Table S11). The addition of oceanic boundlessness in the second model did not lead to a significant increase in R^2^ (p = .053) and did not significantly contribute to explaining the variance in LCI scores. Therefore, a mediation analysis was not run.

| Table S11. Hierarchical regression analysis predicting LCI Self-Acceptance at day 85 from associated 5D-ASC subscales | | |
| --- | --- | --- |
|  | Model 1 | Model 2 |
| Group |  |  |
| 10mg vs placebo | 0.62* | 0.37* |
| 25mg vs placebo | 0.50* | 0.22 |
| 5D-ASC oceanic boundlessness |  | 0.31 |
| R^2^ | 0.27* | 0.31* |
| Figures shown are standardised coefficients. Group was represented as two dummy variables. * *p* <.05 | | |

**LCI Spirituality Day 8**

A hierarchical regression was run to examine whether 5D-ASC oceanic boundlessness predicted LCI spirituality at day 8, with group in the first model, and 5D-ASC oceanic boundlessness added in the second model (Table S12). The addition of oceanic boundlessness in the second model led to a significant increase in R^2^ (p < .001) and significantly contributed to explaining the variance in LCI scores. To explore a possible mediational effect of oceanic boundlessness, a mediation analysis was run with group as the independent variable, 5D-ASC oceanic boundlessness as the mediator, and LCI spirituality at day 8 as the dependent variable. Bias corrected bootstrapped CIs for the indirect effects were entirely above zero (b_1_ = 0.37 [0.16 – 0.61], b_2_ = 0.43 [0.20 – 0.68]), indicating a significant mediation effect of group on LCI Spirituality at day 8 through oceanic boundlessness. The direct effect of group in the presence of the mediator was not significant (c_1_ = 0.14, c_2_ = -0.02, p = .190), indicating full mediation.

| Table S12. Hierarchical regression analysis predicting LCI Spirituality at day 8 from associated 5D-ASC subscales | | |
| --- | --- | --- |
|  | Model 1 | Model 2 |
| Group |  |  |
| 10mg vs placebo | 0.58* | 0.16 |
| 25mg vs placebo | 0.47* | -0.02 |
| 5D-ASC oceanic boundlessness |  | 0.55* |
| R^2^ | 0.23* | 0.35* |
| Figures shown are standardised coefficients. Group was represented as two dummy variables. * *p* <.05 | | |

**LCI Spirituality Day 85**

A hierarchical regression was run to examine whether 5D-ASC oceanic boundlessness predicted LCI spirituality at day 85, with group in the first model, and 5D-ASC oceanic boundlessness added in the second model (Table S13). The addition of oceanic boundlessness in the second model led to a significant increase in R^2^ (p = .030) and significantly contributed to explaining the variance in LCI scores. To explore a possible mediational effect of oceanic boundlessness, a mediation analysis was run with group as the independent variable (using indicator coding, with placebo as the reference group), 5D-ASC oceanic boundlessness as the mediator, and LCI Spirituality at day 85 as the dependent variable. Bias corrected bootstrapped CIs for the indirect effects included zero (b_1_ = 0.32 [-0.01 – 0.68], b_2_ = 0.38 [-0.01 – 0.79]), indicating no significant mediation effect.

| Table S13. Hierarchical regression analysis predicting LCI Spirituality at day 85 from associated 5D-ASC subscales | | |
| --- | --- | --- |
|  | Model 1 | Model 2 |
| Group |  |  |
| 10mg vs placebo | 0.37* | 0.06 |
| 25mg vs placebo | 0.34* | -0.02 |
| 5D-ASC oceanic boundlessness |  | 0.39* |
| R^2^ | 0.10* | 0.16* |
| Figures shown are standardised coefficients. Group was represented as two dummy variables. * *p* <.05 | | |
